# Optimization of Bioactive Phenolics Extraction and Cosmeceutical Activity of Eco-Friendly Polypropylene-Glycol–Lactic-Acid-Based Extracts of Olive Leaf

**DOI:** 10.3390/molecules27020529

**Published:** 2022-01-14

**Authors:** Marijan Marijan, Anamarija Mitar, Lejsa Jakupović, Jasna Prlić Kardum, Marijana Zovko Končić

**Affiliations:** 1 Faculty of Pharmacy and Biochemistry, University of Zagreb, Marulićev trg 20, HR-10000 Zagreb, Croatia; mmarijan@pharma.hr (M.M.); ljakupovic@pharma.hr (L.J.); 2 Faculty of Chemical Engineering and Technology, University of Zagreb, Marulićev trg 20, HR-10000 Zagreb, Croatia; anamarija.mitar@gmail.com (A.M.); jprlic@fkit.hr (J.P.K.)

**Keywords:** anti-aging, antioxidant, cosmetic, mixture design, oleuropein, olive leaf, optimization, phenol

## Abstract

Olive leaf is a rich source of phenolic compounds with numerous activities related to skin health and appearance. In this study, a green extraction method was developed using eco-friendly solvents: polypropylene glycol (PPG), lactic acid (LA), and water. The optimal extraction conditions were established, including solvent, extraction time, technique (magnetic stirrer vs. ultrasound-assisted extraction), and herbal material/solvent ratio. The composition of the solvent mixture was optimized using a mixture design. The content of phenolic compounds, including oleuropein and verbascoside, was determined using high-performance liquid chromatography (HPLC) and spectrophotometric methods. Using different extraction conditions, three extracts were prepared and their phytochemical compositions and antioxidant and skin-related bioactivities were investigated. The extracts were excellent inhibitors of elastase, collagenase, tyrosinase, and lipoxygenase. The best activity was shown by the extract richest in phenolics and prepared using magnetic-stirrer-assisted extraction for 20 min, with 0.8 g of herbal material extracted in 10 mL of PPG/LA/water mixture (28.6/63.6/7.8, *w*/*w*/*w*), closely followed by the extract prepared using the same extraction conditions but with 0.42 g of herbal material. The investigated PPG/LA/water mixtures contributed to the overall enzyme-inhibitory activity of the extracts. The prepared extracts were appropriate for direct use in cosmetic products, thus saving the time and energy consumption necessary for the evaporation of conventional solvents.

## 1. Introduction

Olive (*Olea europaea* L., Oleaceae) is a small tree traditionally grown in the Mediterranean Basin, mostly used for the production of oil from the fruit of the plant. However, the olive oil industry generates high amounts of waste, particularly during the agricultural phase, which has polluting effects on soil and water. One of the most prominent olive byproducts is olive leaf, which represents about 5% of the weight of the olives [1]. Olive leaf is a traditional herbal remedy, commonly utilized as a therapy for numerous chronic conditions [2]. Its prominent biological effects include anti-atherosclerotic, hypoglycemic, cardioprotective activity, and radioprotective activity, as well as anti-fungal, anti-proliferative, and cytotoxic effects [3]. The major phenolic constituent of olive leaf is a bitter secoiridoid, oleuropein, widely recognized as an excellent antioxidant with outstanding anti-inflammatory properties [4]. Furthermore, oleuropein shows antimicrobial activity versus bacteria, fungi, yeasts [4], and other parasites [5]. In addition, numerous studies show that the phenolic compounds present in olive leaves, and oleuropein in particular, are associated with antihypertensive, hypoglycemic, hypocholesterolemic and cardioprotective activity [6]. Oleuropein can also inhibit endothelial activation, monocyte cell adhesion, and platelet aggregation [2]. Commercially available olive-leaf extracts, standardized for oleuropein content, are widely utilized to enrich various products with enhanced biological and biomedical properties [7]. Verbascoside, another phenolic antioxidant based on hydroxytyrosol, is the main representative of hydroxycinnamic derivatives in olive leaf [8]. Verbascoside has many pharmacologically favorable activities for human health, including anti-inflammatory, antioxidant, and antineoplastic properties, in addition to wound-healing and neuroprotective properties [9]. In addition, olive leaf contains other valuable phenolic and other ingredients such as flavonoids (e.g., rutin and luteolin), phenylethanoids (e.g., hydroxytyrosol), lignans (e.g., syringaresinol), and iridoids (e.g., loganic acid) [1].

In addition to their utilization in food, plant phenolics are increasingly used in cosmetic products. The demand for natural and organic cosmetics is growing rapidly every year, which increases interest in researching herbal extracts that contain biologically active ingredients with the potential to prevent and delay skin aging [10]. However, to incorporate phenolics and other bioactive plant metabolites into cosmetic formulations, they must be effectively extracted from dried or fresh plant material, preferably using “green” extraction techniques and solvents [11]. One of the solvents that can easily fit into this category is polypropylene glycol (PPG), a non-flammable, non-toxic liquid, generally recognized as safe (GRAS). PPG is a biocompatible [12] and biodegradable [13] polymer freely soluble in water [14]. It is widely used by the chemical, food, and pharmaceutical industries [12]. Despite its non-toxicity and humectant properties, as well as the fact that it is used in cosmetic products [14], the use of PPG mixtures for extraction of bioactive natural compounds is currently underexplored. However, its use in biphasic systems has shown that it is an excellent solvent for the extraction of natural and synthetic dyes [12,15].

Lactic acid (LA) is an alpha-hydroxy acid (AHA) of natural origin and a well-known part of the skin’s natural moisturizing complex. LA contributes to the cell cycle in human keratinocytes and is a common product of bacteria in the skin [16]. It is a GRAS substance and a common ingredient in cosmetic products. In addition to its pH-regulating properties and antimicrobial activity, it induces skin hydration and renewal [16]. Clinical and histologic evidence indicates that the topical application of LA is effective for depigmentation and for improving the surface roughness and mild wrinkling of the skin caused by environmental photo-damage [17]. In addition, the presence of weak acids such as LA may prevent the alkaline-conditions-induced degradation of some flavonoids during the extraction process [18]. For example, LA-containing solvents have been demonstrated to preserve the antioxidant activity of arecanut seed extracts [19]. Finally, LA was among the most efficient components of the specially designed deep eutectic solvents that contributed to the recovery of oleuropein and other high-added-value products from olive leaf [20].

Various formulations of olive-leaf extracts are routinely used in the cosmetic industry [21]. Oleuropein, for example, exhibits effects beneficial for the skin, such as anti-aging and anti-inflammatory properties [22]. Verbascoside has shown promising activity as a potential therapeutic agent for the treatment of atopic dermatitis [23]. Bearing in mind the toxic effect of olive leaf on the environment, as well as the widespread cosmetic use and environmentally friendly nature of PPG and LA, the aim of this work was to optimize the PPG/LA/water extraction of olive-leaf phenolics and to investigate the antioxidant and skin-anti-aging-related activity of the prepared extracts.

## 2. Results

### 2.1. Optimization of the Solvent Composition According to the Mixture Design

In order to determine the optimal mixture of PPG, LA, and water for the extraction of biologically active components from olive leaves, an IV-optimal mixture type of design of experiment (DOE) was used. As water and PPG may be freely used in cosmetic products, their content was set to be between 10% (*w*/*w*) and 89% (*w*/*w*). LA, on the other hand, may display biological activity and different responses depending on its concentration in the cosmetic product. LA and other AHAs may be beneficial to the skin at low concentrations due to epigenetic modifications of the inflammasome complex. Topical application of LA and other AHAs may disrupt the cohesion of the corneocytes in the skin barrier, a feature useful in so-called chemical peelings. However, the application of high concentrations may also result in skin irritation [16]. In order to prevent the possible side effects of high LA concentration, its content in the final solvent extraction mixture was set to be between 1% (*w*/*w*) and 10% (*w*/*w*).

The main distinction between the mixture DOE and other types of DOEs is that in the former, the input variables are proportionate to the amounts of the components in the mixture. Furthermore, in the mixture DOE, the measured response is assumed to depend only on the relative proportions of the ingredients or components in the mixture [24]. Using the appropriate ternary mixtures, sixteen solvents with different physicochemical properties (Table S1) (see Supplementary Materials) and the corresponding extracts were prepared, and the concentrations of target phenolics were determined (Table 1).

From the data presented in Table 1 it may be noted that the concentration of the target compounds depended greatly on the extraction solvent composition. For example, the TP values ranged from to 1.547 mg/mL to 5.863 mg/mL, representing an almost four-fold increase in the reaction yield. In general, it may be concluded that a lower content of PPG contributed positively to the TP. For example, mixtures with more than 80 *w*/*w* % of PPG showed a viscosity two orders of magnitude higher than solvents with the lowest PPG content (10%, *w*/*w*), Table S1. It can be assumed that improved hydrodynamic conditions, which can be achieved in low-viscosity solvents, contribute to the extraction efficiency. Conventional extraction methods usually use large quantities of organic solvents that are not appropriate for direct dermal use. Water is among the few conventional solvents that may be used in cosmetic formulations. In order to compare the efficacy of the newly prepared PPG/water/LA solvents with conventional water extraction, an aqueous extract was prepared using MSAE under the same extraction conditions. The TP content of this extract was 1.05 mg/mL, which was notably lower than the TP content of any of the extracts presented in Table 1. Moreover, the TP of aqueous extracts was as much as 5-fold lower than the TP of the extracts richest in phenolic compounds (e.g., runs 2, 3, 8 10, and 12). The content of oleuropein ranged from 0.552 to 1.574 mg/mL, representing a three-fold increase in its content depending on the extraction conditions. Depending on the extract, oleuropein accounted for approximately 30% of the TP. Similar to TP, it seems that PPG and oleuropein content were inversely related.

Using a multiple regression analysis, a model was fitted to the experimental results. Two polynomial equations that explain the relationship between the responses and the independent variables were produced as a result. The relationship between the extraction conditions (as actual components, given as ratios) with TP and oleuropein content are presented in Equations (1) and (2) below.

TP (mg/mL) = 1.14 × X_1_ + 18.42 × X_2_ − 604.21 × X_3_ + 13.13 × X_1_ × X_2_ + 686.95 × X_1_ × X_3_ + 683.52 × X_2_ × X_3_, (1)

Oleuropein (mg/mL) = 0.35 × X_1_ + 0.29 × X_2_ − 49.60 × X_3_ + 2.17 × X_1_ × X_2_ + 52.97 × X_1_ × X_3_ + 64.54 × X_2_ × X_3_, (2)

To determine the statistical significance of the obtained models, *p*-values and *F*-tests were utilized (ANOVA, Table 2). The calculated *F*-values of both models were higher than 5, while the *p*-values for the models were lower than 0.05. In contrast, *p*-values for the lack of fit in the models were higher than 0.05. This showed the significance of the models, as well as their suitability for the interpretation of the experimental data. The determination coefficients (*R*^2^) for TP and oleuropein extraction were relatively high (0.8446 and 0.9235), showing that the observed values were well described by the selected optimization models. The predicted *R*^2^ was in good agreement with the adjusted values, additionally confirming the ability of the models to optimize and predict the selected responses.

The calculated values of the independent variables best suited to producing the extracts with the highest TP and oleuropein concentration are presented in Table 3. Based on the obtained results, the corresponding extracts were prepared, and the contents of the target compounds were determined. The predicted and experimentally obtained values shown corresponded well in both extracts, with the deviations being less than 2%. This additionally confirms the suitability of the models for predicting the properties of PPG/water/LA mixtures for dissolving oleuropein and other olive-leaf phenolics. Based on the obtained results, the corresponding solvents were prepared and used in further studies.

### 2.2. Selection of the Extraction Method

Due to the numerous beneficial biological activities they display, plant phenolics are among the most researched and appreciated secondary plant metabolites. However, due to their sensitivity to oxidation, the conditions used for their extraction may strongly influence not only the phytochemical composition of prepared extracts but also their pharmacological activity [25]. In order to obtain a feasible and scalable extraction process, the objective of the further extraction optimization was to find the best extraction conditions with respect to not only the amount of the extracted target phenolics but also the time, energy, and herbal material consumption. In order to observe how solvents with different compositions and physicochemical properties affect the extraction efficiency, two solvent systems (Table 3) found to be the best suited for the extraction of total olive phenolics (solvent A) and oleuropein (solvent B) were chosen for further optimization with regard to extraction duration, technique, and herbal material/solvent ratio (HM/SR).

**Figure 1:**
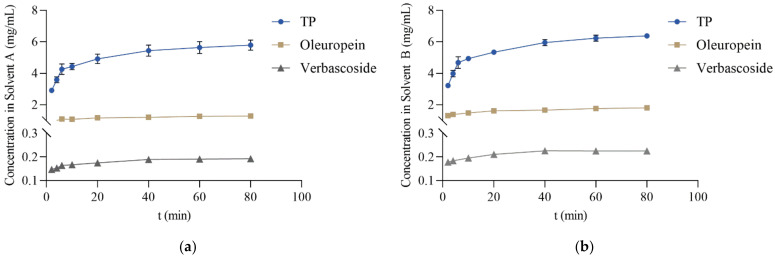
Kinetics plots of total phenolic content (TP), oleuropein, and verbascoside extraction from olive leaf using the solvents (**a**) A and (**b**) B.

The first step in this process was to determine the extraction duration that would enable a good yield of the target compounds while simultaneously making the extraction process time-efficient and consequently energy-effective. Hence, the kinetics of TP, verbascoside, and oleuropein extraction using the two extraction techniques were studied (Figure 1).

As may be observed from the slope of the kinetics curves, most of the target phenolics were extracted in the first 20 min of the extraction. After that time and up to 80 min of extraction, there were only relatively small increases in the concentrations of the target compounds in solvents A (17.8% for TP, 9.9% for oleuropein, and 9.7% for verbascoside) and B (19.3% for TP, 11.6% for oleuropein, and 6.6% for verbascoside). As the four-fold increase in the extraction duration and the corresponding energy consumption of the extraction process was not justified by a relatively small increase in the concentration of released active substances, a 20 min extraction time was selected as the optimal extraction duration and used in the following experiments.

In the following steps, the efficacy of two different extraction techniques, i.e., magnetic-stirring-assisted extraction (MSAE) and ultrasound-assisted extraction (UAE) was compared to assess their suitability for the extraction of olive phenolics in two different solvents. Furthermore, the relationship between the concentration of extracted phenolics and the HM/SR was observed. MSAE and UAE are among the simplest and the most commonly utilized extraction techniques. The main difference between these two techniques is the mechanism of active substance extraction from the plant cells. Ultrasound waves in UAE cause fragmentation of the cell wall and rinse the cell substances once the walls are fragmented [26]. This allows for an easier approach of the solvent to the cell and increases the mass transfer [27]. MSAE, on the other hand, enables high-speed stirring, which reduces the concentration gradient in the solution surrounding the plant material particles, allowing for faster access of the solvent molecules to the particles and accelerating the diffusion of the active substances into the solution [28].

**Figure 2:**
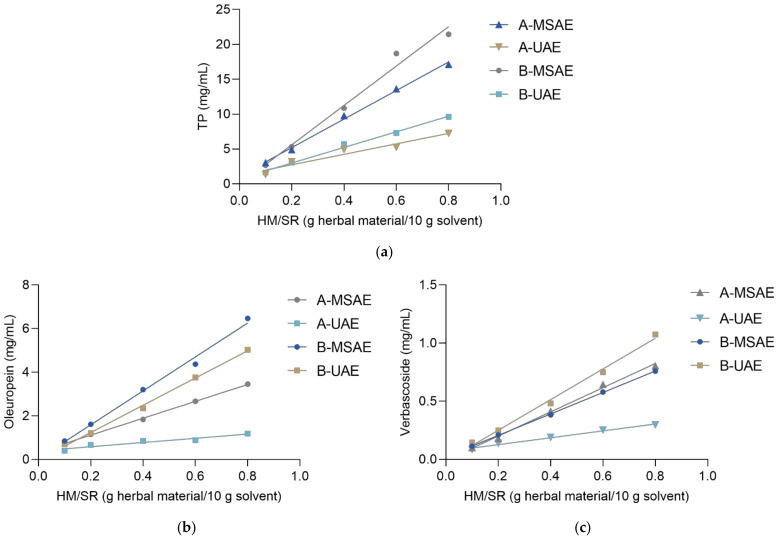
The relationship between the HM/SR (herbal material/solvent ratio) and the concentration of (**a**) TP, (**b**) oleuropein, and (**c**) verbascoside in the extracts. A, B denote the extraction solvent; UAE, MSAE denote the extraction technique.

Although UAE is often regarded as a fast, simple, and cost-efficient extraction technique, characterized by high reproducibility [29], the data for TP and oleuropein extraction presented in Figure 2 clearly demonstrate that extraction using MSAE showed superior results in comparison with UAE. This observation can be interpreted as indicating the possible degradation of sensitive phenolic compounds of the olive leaf when exposed to ultrasonic waves [30]. On the other hand, the verbascoside extraction displayed a different trend compared to oleuropein, and UAE was the method of choice for the extraction of verbascoside from olive leaf. However, the concentration of verbascoside was relatively low compared to oleuropein and only slightly affected the TP. For example, after 20 min of extraction (Figure 2), the concentration of verbascoside reached only 4.0% of the TP. Although at low HM/SRs the TP were slightly better dissolved in the more polar solvent A, at higher HM/SRs the more apolar solvent B was more appropriate for the extraction of all olive phenolics. It was found that the relationships between the concentrations of the target phenolics and the HM/SR were linear with good determination coefficients (*R*^2^ = 0.9163–0.9976). The equations and the corresponding coefficients of determination are listed in Table S2 (see Supplementary Materials). Thus, it was concluded that solvent B combined with MSAE should be the method of choice for the overall extraction of olive-leaf phenolics.

As the B-MSAE method was the most successful in dissolving the majority of the target compounds, additional experiments were performed to observe whether saturation of the extraction solution would occur at high HM/SRs. Thus, experiments where 1.0–1.6. g of olive leaf was extracted using B-MSAE were also performed. It was interesting to observe that TP, oleuropein, and verbascoside concentrations increased linearly, even when the ratio of HM/SR was as high as 1.6 g/10 g (Table S3) (see Supplementary Materials). While this is the first report on the olive phenolics extraction yield using LA/PPG-based solvents, the presented findings are in accordance with previous studies where LA enhanced the extraction yield and stability of extracts of phenolic antioxidants of olive leaf [20] and Mediterranean plants [31] in cyclodextrin-based deep eutectic solvents. Furthermore, LA contributed to the stability of glycerol extracts of *Echinacea purpurea* [32].

**Figure 3:**
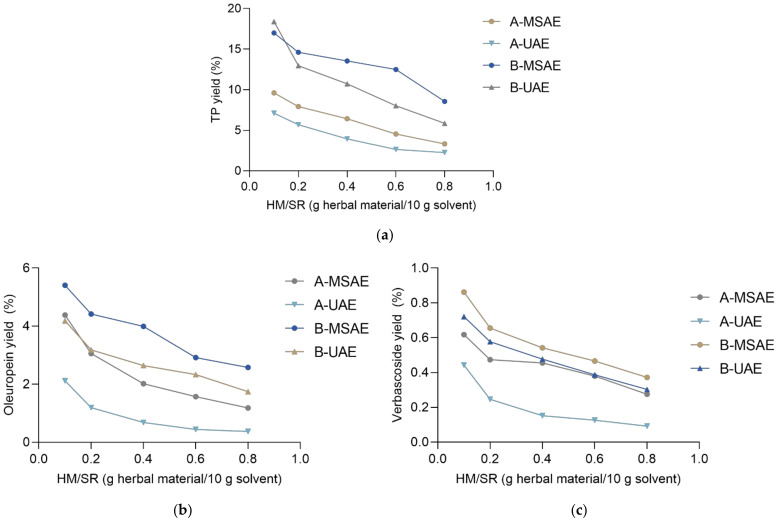
The relationship between the HM/SR (herbal material/solvent ratio) and the yield (%) for (**a**) total phenol (TP), (**b**) oleuropein, and (**c**) verbascoside content. A, B denote the extraction solvent; UAE, MSAE denote the extraction technique.

During the extraction process, the plant material swells, soaking up water, and thus effectively reduces the amount of extract obtained [33]. Thus, despite the high concentration of the target compounds, the small amount of the extract obtained at high HM/SRs inevitably leads to a low total weight of the extracted compound. At this ratio, however, the volume (Table S4) (see Supplementary Materials) of the prepared extracts was so low that the extraction process was essentially meaningless. With that in mind, the yield of the extracted compounds was also observed (Figure 3) and recorded as the weight of the target phenolics per weight of the herbal material. As expected, the best yield was achieved at the lowest HM/SRs, because those conditions enabled exhaustive extraction of the herbal material and thus the highest extraction efficiency. The curves representing the relationship between the yield and HM/SR were second-order polynomial (quadratic) equations and showed good to excellent relationships, with correspondingly high *R*^2^ values (Table S5) (see Supplementary Materials).

### 2.3. Preparation and Phytochemical Composition of the Extracts Used for Bioactivity Testing

Based on the results of the previous experiments, three extracts were prepared for the biological studies. The first extract, B042M, was prepared according to the desirability function for simultaneously having the highest yield and content of the target variables. This parameter was selected in order to increase the environmental end economical acceptability of the extraction (e.g., to extract as much as possible of the target compound using the least amounts of the plant material and the solvent). On the other hand, B08M was the extract with the highest concentration of the target compounds (TP and oleuropein). Finally, A08U was selected as an example of an extract prepared using a different method and a solvent of different polarity. The content of the selected groups of phenolic compounds in the prepared extracts are presented in Table 4.

As anticipated, the highest concentration of almost all the selected groups of phenolics was found in sample B08M; the only exception was the verbascoside content, which was slightly higher in A08U.

### 2.4. Antioxidant Activity

Botanicals, as active ingredients, represent one of the largest categories used in dermatology. One of the botanicals’ important characteristics is their antioxidant activity and the related ability to counteract oxidative stress and impede activation of proteolytic enzymes. This leads to the prevention of age- and environment-related skin changes and consequently to the prevention of skin aging. The antioxidant activity of the extracts prepared in this work was investigated using two methods: 2,2-diphenyl-1-picrylhydrazyl (DPPH) radical scavenging activity and β-carotene–linoleic-acid assay. As it was not possible to evaporate the PPG-containing solvents, the unit for the IC_50_ values of the extracts is expressed as μL of the extract per mL of the reaction solution (μL extract/mL). When examining the antioxidant activity of the extracts, the activity of the standard inhibitor, butylated hydroxyanisole (BHA), was also assessed and expressed as μg/mL. It is important to note that a direct comparison of the extracts’ IC_50_ values with the IC_50_ value of the standard was not possible due to different measurement units. However, as the IC_50_ value of the BHA was numerically equal to the IC_50_ value of the 1 mg/mL BHA solution, the activity of the standard is reported for informative purposes and qualitative comparison.

**Figure 4:**
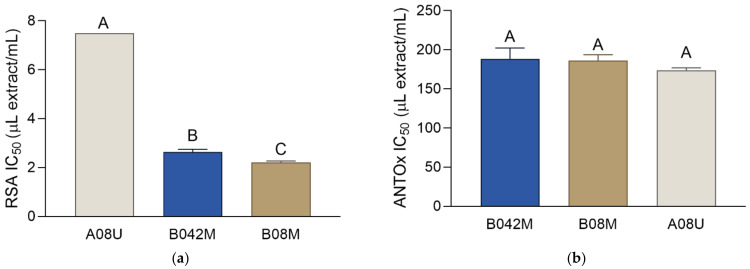
Antioxidant activity: (**a**) radical scavenging activity (RSA) and (**b**) antioxidant activity in β-carotene–linoleate assay (ANTOx) of the extracts (A08U, B042M, B08M). The activities are presented as IC_50_ values ± SD. Different uppercase letters indicate statistically significant differences (*p* < 0.05).

The RSA activity of the extracts is presented in Figure 4. The RSA IC_50_ value of the BHA was 10.97 ± 0.18 μg/mL, while the tested extracts demonstrated RSA IC_50_ values lower than 8 μL extract/mL. Numerical comparison of the extracts’ activity with the activity of BHA indicates a rather strong activity in the tested samples. The activity of the two solvents in the DPPH assay was observable and contributed partially to the activity of the extracts. Solvent B exhibited an RSA IC_50_ of 53.69 ± 0.85 μL solvent/mL, while the activity of solvent A was 38.92 ± 3.86 μL solvent/mL. Among the investigated extracts, B08M was the strongest radical scavenger, but its RSA IC_50_ was only slightly lower than the RSA IC_50_ of B042M. In the β-carotene–linoleate assay there was no difference in the activity among the extracts (Figure 4). In addition, their activities were rather similar to the activity of the standard antioxidant BHA, with its ANTOx IC_50_ value of 198.55 ± 8.77 μg/mL. The extraction solvents also showed notable activity in this assay, with ANTOx IC_50_ values of 302.16 ± 41.30 μL solvent/mL and 254.18 ± 10.69 μL solvent/mL for solvents A and B, respectively. This indicates that the activity of the phytochemicals present in the extracts was enhanced by the activity of the solvents’ components. It is interesting to note that the difference in the antioxidant activity between B08M and B042M was either rather small (RSA) or non-significant (ANTOx). As the amount of the herbal material needed for the preparation of B08M was almost twice as high as the amount needed for the preparation of B042M, the use of extra herbal material hardly seems justified in this case. This is further corroborated by the lower yield of B08M. Thus, B042M is the most ecologically acceptable among the prepared extracts.

The DPPH assay is a simple, quick, and commonly used assay for determining the in vitro radical-scavenging or hydrogen-donating activity of natural compounds. In its radical form, DPPH absorbs at 517 nm, but upon reduction with an antioxidant it forms non-radical species and the absorption of the reaction mixture decreases [34]. The basis of the β-carotene–linoleic-acid assay is thermally induced oxidation of the linoleic acid, leading to the generation of hydroperoxide and free radicals. The formed reactive species attack the β-carotene double bonds, disrupting the molecule’s conjugation and leading to the discoloration of the reaction solution [35]. The antioxidant activity of the extracts in the two assays is primarily related to the quenching of the reactive species formed in the two systems by their phytochemical components. In addition, the solvents also played an important role in the overall activity of the extracts. The activity of the solvents in this assay may well be explained by the ability of the LA to act as an antioxidant and scavenge hydroxyl and other free radicals [36,37]. It was previously found that LA-containing deep eutectic solvents combined with cyclodextrins may yield olive-leaf extracts with improved antioxidant properties, compared with other green solvents [20]. To the best of our knowledge, this is the first report showing that the enhanced antioxidant activity of the extracts may also be achieved by using an LA/PPG-based solvent system.

### 2.5. Enzyme Inhibiting Activity

Botanicals can affect the appearance of the skin and reduce skin aging by decreasing the activity of various enzymes in the skin cells and extracellular matrix. They can influence skin’s flexibility, firmness, and pigmentation, as well as its health and function, e.g., by influencing the inflammatory processes that may occur under the influence of endogenous and exogenous factors [38]. To assess how the prepared olive-leaf extracts may affect skin aging, the inhibitory effect on the enzymes elastase, collagenase, tyrosinase, and lipoxygenase (LOX) was investigated. The activity of the respective standard enzyme inhibitors was also assessed. Although similar to the antioxidant activity of the BHA standard, a direct comparison of the extracts’ IC_50_ values with the IC_50_ values of the standards was not possible due to different measurement units, but the activity of the standard is reported for informative purposes and qualitative comparison.

**Figure 5:**
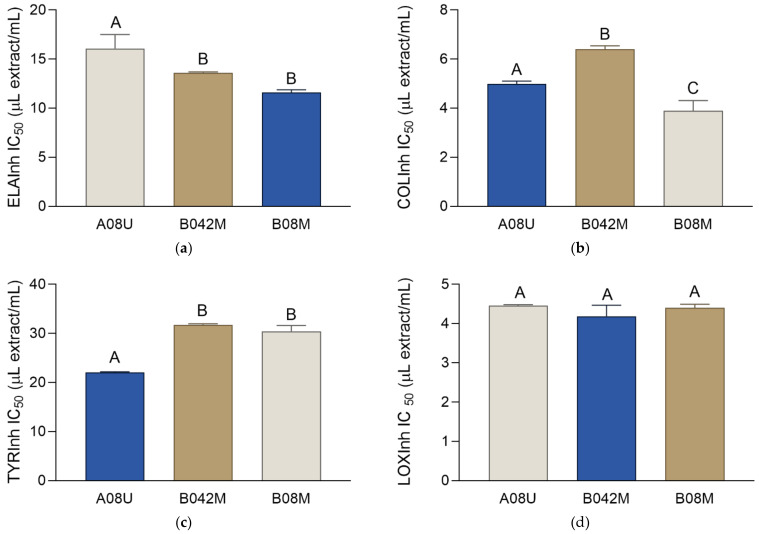
Inhibitory activity of the extracts (A08U, B042M, B08M) on the enzymes that accelerate skin aging: (**a**) elastase (ELAInh), (**b**) collagenase (COLInh), (**c**) tyrosinase (TYRInh), and (**d**) lipoxygenase (LOXInh). The activities are shown as IC_50_ values ± SD. Different uppercase letters indicate statistically significant differences (*p* < 0.05).

Aging is a complex, multi-step process which, among other processes, involves breaking down of collagen and elastin fibers [39]. Collagen and elastin are proteins whose networks make up the majority of the extracellular matrix in many organs, including the skin. While collagen contributes tensile strength to skin, elastin fibers contribute to its extensibility and reversible recoil, allowing it to withstand repeated mechanical deformations without suffering irreversible plastic damage [40]. Thus, collagen and elastin fibers together provide for the skin’s normal strength, hydration, and mechanical properties [39]. The two enzymes that affect skin aging by participating in the decomposition of elastin and collagen are elastase [41] and collagenase [39], respectively. Their increased activity leads to the degenerative changes of the skin that are visible as wrinkles and folds. As shown in Figure 5, the extracts prepared using solvent B were equally strong elastase inhibitors, while A08U displayed weaker activity. The solvents did not show elastase-inhibiting activity, while the activity of the standard, ursolic acid, was 56.98 ± 2.35 μg/mL. Hence, numerical comparison of the IC_50_ values of the extracts and the standard indicates a rather high activity in the prepared extracts. The extracts also demonstrated a notable anti-collagenase activity. As shown in Figure 5, B08M was the strongest collagenase inhibitor, followed by A042M. The COLInh IC_50_ of gallic acid, a standard collagenase inhibitor, was 52.78 ± 7.56 μg/mL. The IC_50_ values of the extracts were numerically lower than that of gallic acid, implying their rather good activity. It is important to note that the anti-collagenase activities of the solvents A and B were 8.30 ± 2.67 μL solvent/mL and 3.65 ± 0.51 μL solvent/mL, respectively. Comparing these values with the COLInh IC_50_ values of the extracts presented in Figure 5, it may be observed that the pure solvents were more active than the extracts. This means that the solvents were the main factors responsible for the activity in this assay, since the phytochemical fraction of the extract was less active than the solvent components.

Tyrosinase is an enzyme that strongly affects skin color by catalyzing the tyrosine oxidation to dopaquinone, which is the first and the rate-limiting step in the biosynthesis of melanin, the substance responsible for the skin pigmentation. Although melanin represents the skin’s natural protection against ultraviolet radiation, high tyrosinase activity and excessive melanin production leads to uneven and aesthetically unacceptable pigmentation [42]. As shown in Figure 5, the most active tyrosinase inhibitor was A08U. While notable, the activity of the extracts was still somewhat weaker than the activity of the standard tyrosinase inhibitor kojic acid, with its TYRInh IC_50_ of 7.43 ± 0.07 μg/mL. The activities of the solvents A and B were 24.64 ± 1.24 μL solvent/mL and 24.13 ± 0.15 μL solvent/mL. Similar to the collagenase inhibition, these high TYRInh IC_50_ values indicate that the solvents were the main factors responsible for the activity in this assay.

As well as being due to exposure to UV radiation, uneven pigmentation may occur as a consequence of skin inflammation. Anti-inflammatory activity is considered as one of the most important aspects of the cosmeceutical activities of plants, their extracts, and the products that contain them. Skin inflammation can lead to redness, swelling, or disturbed skin physiological functions [36], which are all processes that strongly affect the skin’s health and appearance. In this study, the anti-inflammatory activity of the extracts was investigated using the LOX-inhibiting assay. LOX isozymes play a role in the barrier function, inflammatory skin diseases, and wound healing, as well as in the modulation of epithelial proliferation and differentiation [43]. The results of the LOX-inhibiting activity (Figure 5) show that the activities of the extracts did not differ among themselves. The standard inhibitor, nordihydroguaiaretic acid (NDGA), showed a numerically lower LOXInh IC_50_ (2.34 ± 0.07 μg/mL) than the extracts. The activities of solvents A and B were 4.06 ± 0.47 μL solvent/mL and 7.69 ± 0.81 μL solvent/mL, respectively, indicating that the activity of the extracts was again at least partly aided by the activity of the solvents. Similar to antioxidant activity determination, in most of the performed enzymatic assays, the difference in the activity between B08M and B042M was non-significant. Hence, B042M was the most ecologically acceptable among the prepared extracts.

The phenolics and secoiridoids found in the extracts of olive exhibit anti-aging properties by showing antioxidant activity and the capacity to inhibit the enzymes related to skin aging. For example, oleuropein and its metabolite hydroxytyrosol are elastase and collagenase inhibitors that may permeate skin and show synergistic cellular antioxidant effects in human skin fibroblasts [44]. While non-flavonoid olive phenols are not able to significantly inhibit tyrosinase, flavonoids present in the olive-leaf extracts, such as rutin, may display such activity [45]. Previously investigated hydroalcoholic olive-leaf extracts were able to efficiently inhibit elastase and hyaluronidase [1]. Furthermore, olive-flower extracts were found to inhibit the action of elastase and collagenase, while tyrosinase was inhibited to a much lesser extent, both by the extracts and the isolated compounds [46]. However, these extracts were prepared using hydroalcoholic solutions [46] that need to be evaporated prior to incorporation into cosmetic products; a process that consumes a considerable amount of time and energy rendering it ineffective from the economic and ecological point of view. The extracts prepared in this study have the advantage of being prepared using solvents that may not only be incorporated into final product but can actively contribute to the overall activity of the prepared extracts. For example, the strong activity of the solvents in the collagenase and tyrosinase inhibition assays is not surprising, because the activity of both collagenase [47] and tyrosinase [48] is pH-dependent, with a rapid decrease below pH 5.5. As the LA-rich solvents prepared in this study typically had a pH below 3 (Table S1), they would be stronger tyrosinase inhibitors than the extracts, where a part of the LA molecules is displaced by the phytochemical components. In addition, due to their LA content and the consequent peeling effect [16], the solvents prepared within this study may actively contribute to the permeation of the extracts’ phytochemical components into the skin. Unlike elastase-, collagenase- and tyrosinase-inhibiting properties, the anti-LOX activity of the olive-leaf extracts has not been previously reported. However, it has been found that some of the olive phytochemicals may display such activity. Oleacein, a dialdehyde and a known LOX, may be one of the substances contributing to the observed activity of the extracts, along with olive phenolics and secoiridoids [49].

## 3. Materials and Methods

### 3.1. Chemicals

In this study, BHA, β-carotene, DPPH, Folin–Ciocalteu reagent, polyethylene glycol 3350 (PEG), *N*-succinyl-tri-*L*-alanine-4-nitroanilide (SANA), verbascoside (Sigma-Aldrich, St. Louis, MO, USA), collagenase, elastase (Alfa Aesar, Haverhill, MA, USA), PPG 425 (A&C, Saint-Laurent, QC, Canada), (*S*)-LA (Merck, Kenilworth, NJ, USA), oleuropein (Fluka, Charlotte, NC, United States), NDGA (Acros Organics, Geel, Belgium), LOX, and linoleic acid (TCI, Tokyo, Japan) were used. The purity of the standards was ≥ 98%. Methanol was of HPLC grade.

### 3.2. Plant Material

Olive leaf (“oblica” cultivar) was collected on the Adriatic island of Ugljan (44°5′N, 15°10′E), Croatia. After grinding, the plant material was sieved in a Retsch AS 200 shaker (Düsseldorf, Germany), using the 800 μm sieve for 3 min. Sampling was performed by quarternation [50]. The sieved herbal material was divided into four equal piles, and two opposite piles were removed. This procedure was repeated until a representative sample weighing about 100 g was obtained. The remaining material was used for the preparation of the extracts.

### 3.3. Selection of Extraction Solvent

Selection of the extraction solvent was performed according to the IV-optimal mixture design, using Design-Expert software version 8.0.6 (Stat-Ease, Minneapolis, MN, USA). The independent variables were as follows (the content is expressed as the weight ratio, while the values in the brackets represent the minimum and the maximum values, respectively): PPG (0.100–0.890 g), water (0.100–0.890 g), and LA (0.010–0.100 g). The dependent variables were the phenolic compounds (oleuropein and total phenols). For the preparation of the extracts according to the optimal mixture design, 0.2 g of powdered olive leaf was added to 10 g of the solvent. The mixtures were stirred at 25 °C for 3 h at 300 rpm using a magnetic stirrer (2mag MIX 15 eco multi-position, Munich, Germany). After extraction, the mixture was filtered using folded filter papers (Filtrak, 80 g/cm^3^, grade 6). The remaining solutions were immediately used for the determination of phytochemical composition. Based on the maximum content of the selected dependent variables (TP and oleuropein content) in the prepared extracts, two different ternary solvent mixtures, solvent A (10% PPG, 89% water, 1% LA) and solvent B (28.6% PPG, 63.6% water, 7.8% LA), were chosen for further optimization of the extraction procedure, including extraction duration, technique, and optimal HM/SR.

### 3.4. Kinetic Measurements

In order to define the optimal extraction duration, MSAE kinetic measurements were performed. In 10 g of solvent, 0.2 g of olive leaf was suspended and mixed at 25 °C and 300 rpm. For each experiment, a separate suspension was prepared. The solutions were analyzed at the following times: 2, 4, 6, 10, 20, 40, 60, and 80 min.

### 3.5. Comparison of UAE and MSAE

Varying weights of the herbal material (0.2–0.8 g) were suspended in 10 g of each of the two ternary solvent mixtures (A or B) and extracted using either MSAE or UEA. MSAE was performed at 25 °C and 300 rpm, while UAE was performed in an ultrasonication bath (Bandelin SONOREX Digital 10 P DK 156 BP) at 25 °C and a power of 360 W. The yield of the target compounds was calculated as described in Equation (3):

Yield (%) = (γ × V)/w × 100(3)

where γ is the target compound mass concentration, V is the volume of the prepared extract, and w is the weight of the plant material used for the extraction.

### 3.6. Preparation of the Extracts for Biological Testing

Based on the results of the previous experiments, three extracts were prepared for the biological studies. For the preparation of A08U, 0.8 g of olive leaf was suspended in 10 g of solvent A and extracted at 25 °C using an ultrasonication bath operating at a power of 360 W. For the preparation of B08M and B042M, either 0.8 g (B08M) or 0.42 g (B042M) of olive leaf was suspended in 10 g of solvent B. The extraction was performed using MSAE at 25 °C and 300 rpm for 20 min. After the extraction, all the mixtures were filtered, and the remaining solutions stored at −20 °C.

### 3.7. HPLC-DAD Determination of Oleuropein and Verbascoside Concentration

The quantification of oleuropein and verbascoside content was performed using an HPLC instrument with an Eclipse XDB-C18 column, equipped with an autosampler and a DAD detector (Agilent 1200 series, Agilent Technologies, Santa Clara, CA, USA), using the modified European Pharmacopoeia method [51]. Prior to analysis, the samples were filtered through a 25 mm, 0.45 µm disposable syringe filter (CHROMAFIL Xtra PTFE, Macherey-Nagel, Düren, Germany). The injection volume was 1 μL. Separation was performed at room temperature, using a flow rate of 1 mL/min. Mobile phase A was 1% glacial acetic acid and mobile phase B was methanol, used according to the following protocol: 0–5 min 15% B, 5–12 min 40% B, and 12–15 min 15% B. The peak assignment and identification were based on the comparison of retention times and the UV/Vis spectra of the peaks in the sample chromatograms with those of the standards. Calibration curves and limits of detection (LD) and quantification (LQ) for oleuropein (y = 629155x + 32, *r*^2^ = 0.9999, LD = 0.0079 mg, LQ = 0.0239 mg) and verbascoside (y = 515085x − 14, *r*^2^ = 0.9999, LD = 0.0050 mg, LQ = 0.0153 mg) (y = area under curve in chromatogram and x = weight of the standard in mg) were determined according to [52].

### 3.8. Spectrophotometric Determination of Total Phenolic Content

Total phenolic content (TP) was determined using the modified Folin–Ciocalteu method, by mixing 80 μL of Folin–Ciocalteu reagent, 80 μL of 10% sodium carbonate solution and 80 μL of the extract solution [53]. After 1 h, the absorbance at 700 nm was measured (FLUOstar Omega, BMG Labtech, Ortenberg, Germany). The TP concentration was calculated from the calibration curve of oleuropein and expressed as mg of oleuropein per mL of extract (oleuropein equivalents). Additionally, the calibration curve of gallic acid was also constructed. By dividing the slope of the oleuropein calibration curve (48.479) with the slope of the gallic acid calibration curve (6.869), it may be calculated that the results expressed as oleuropein equivalents were 7.057-fold (48.479/6.869) higher than those expressed as gallic acid equivalents.

### 3.9. Spectrophotometric Determination of Total Flavonoid Content

Total flavonoid content (TF) was determined using modified Folin–Ciocalteu colorimetric method [54], by mixing 120 μL extract solution and 120 μL of 0.2% AlCl_3_ solution. After 1 h, the absorbance at 420 nm was measured. TF was expressed as mg of quercetin per mL of extract.

### 3.10. Spectrophotometric Determination of Total Phenolic Acid Content

Total phenolic acid content (TPA) was determined using the modified method described by Nicolle et al. [55], where 50 μL of each reagent (0.5 M HCl, nitrite–molybdate reagent and 8.5% NaOH) was added to 100 μL of extract solution. TF was expressed as mg of caffeic acid per mL of extract.

### 3.11. Radical Scavenging Activity

An aliquot of the extract solution (130 μL) and 70 μL of DPPH solution (0.204 mg/mL) were mixed, and the absorbance at 545 nm recorded after 30 min. Methanol (130 μL) was applied as the negative control and BHA (130 μL) as the positive control. Antiradical activity was calculated according to Equation (4):

RSA (%) = (A_0_ − A_s_)/A_0_ × 100 (4)

where A_0_ is the absorbance of the negative control and As is the absorbance of the sample. RSA IC_50_ was calculated as the concentration of extract that scavenged 50% of DPPH free radicals, expressed as μL of extract/mL of solution (μL extract/mL).

### 3.12. Antioxidant Activity in β-carotene–linoleic-acid Assay

The reaction mixture for the determination of antioxidant activity in the β-carotene–linoleic-acid assay (ANTOx) [56] consisted of 200 μL of the aqueous emulsion with β-carotene (6.7 μg/mL), linoleic acid (0.7 mg/mL), and Tween 40 (6.7 mg/mL), together with 50 μL of the extract solution in methanol, heated to a temperature of 50 °C. Reaction mixtures containing methanol or BHA solution instead of the extract served as the negative and positive controls, respectively. The ANTOx was calculated based on the absorbance recorded after 0 min and 60 min, as shown in Equation (5):

ANTOx (%) = (A_s60_ − A_c60_)/(A_c0_ − A_c60_) × 100 (5)

where A_c0_ and A_c60_ are the absorbances of the water control after 0 and 60 min, respectively, while A_a60_ is the absorbance of the sample after 60 min. The ANTOx IC_50_ was calculated as the concentration of the extract that protects 50% of the β-carotene present in the solution after 60 min, expressed as μL of extract /mL of solution (μL extract /mL).

### 3.13. Elastase Inhibitory Activity

For the elastase inhibitory activity determination [57], 100 μL of extract solution in Tris-HCl buffer (0.1 M, pH 8.0) was mixed with 25 µL elastase solution (0.052 mg/mL) and left at room temperature for 5 min. Afterwards, a phosphate buffer saline solution of SANA (70 µL, 0.410 mg/mL) was added, and the absorbance was measured at 410 nm after an additional 40 min. The elastase inhibitory activity (ELAInh) was calculated using Equation (6):

ELAInh (%) = (A_0_ − A_s_)/A_0_ × 100 (6)

where A_0_ is the absorbance of the negative control and As is the absorbance of the solution containing the respective extract. Ursolic acid was used as the standard elastase inhibitor. The results were expressed as IC_50_, i.e., the concentration showing ELAInh = 50%.

### 3.14. Collagenase Inhibitory Activity

For the collagenase inhibitory activity determination, 40 μL of extract solution in Tris-HCl buffer (0.1 M, pH 7.5) was mixed with 20 µL collagenase solution (0.1 mg/mL) at room temperature for 5 min. Afterwards, gelatin solution (40 µL, 3.44 mg/mL) in the same buffer was added, and the mixture was incubated for 40 min at 37 °C. Subsequently, 40 µL of stop reagent (25 mM EDTA in 12% (*w*/*w*) aqueous PEG solution) and 90 µL of ninhydrin solution (0.14 M) was added to the reaction mixture and incubated for 15 min at 80 °C. After cooling, 90 µL of citric buffer was added, and the absorbance was measured at 545 nm. The collagenase inhibitory activity (COLInh) was calculated usingEquation (7):

COLInh (%) = (A_0_ − A_s_)/A_0_ × 100 (7)

where *A_0_* is the absorbance of the negative control and *As* is the absorbance of the solution containing the respective extract. Reaction mixtures containing buffer or gallic acid aqueous solutions instead of extract were used as the negative and positive controls, respectively. The COLInh IC_50_ was calculated as the concentration of the extract that inhibited 50% of the collagenase activity, expressed as μL of extract/mL of solution.

### 3.15. Tyrosinase Inhibitory Activity

Extract solution (120 μL) and tyrosinase solution (40 μL), prepared in 16 mM pH 6 phosphate buffer, were mixed. After 5 min in the dark, 60 μL of *L*-3,4-dihydroxyphenylalanine (*L*-DOPA) solution (0.83 mg/mL in phosphate buffer) was added, and the absorbance at 492 nm was measured after 60 min. The negative control contained a PBS buffer instead of the extract solution. The tyrosinase inhibitory activity (TYRInh) was calculated according to Equation (8):

TYRInh (%) = (A_0_ − A_s_)/A_0_ × 100 (8)

where *A_0_* is the absorbance of the negative control and *As* is the absorbance of the solution containing the respective extract. Kojic acid was utilized as the standard tyrosinase inhibitor. The results are expressed as EC_50_, i.e., the concentration showing TYRInh = 50%.

### 3.16. Lipoxygenase Inhibitory Activity

For the LOX inhibitory activity [58], 100 μL of extract solution, 25 μL of LOX solution (0.00667 µL/mL), and 50 μL of phosphate buffer (pH 8, 100 μM) were mixed. After 5 min, 50 μL of linoleic acid in phosphate buffer (pH 8, 100 μM) were added and incubated at 25 °C. After 45 min, the absorbance was determined at 234 nm. Reaction mixtures containing buffer or NDGA solutions rather than extract served as the negative and positive controls, respectively. The LOX inhibitory activity (LOXInh) was expressed as in Equation (9):

LOXInh (%) = (A_c_ − A_s_)/A_c_ × 100 (9)

where *Ac* is the absorbance of the negative control and *As* is the absorbance of the respective extract. The LOXInh IC_50_ was calculated as the concentration of the extract that inhibited 50% of the LOX activity, expressed as μL of extract/mL of solution.

### 3.17. Statistical Analysis

All measurements were performed in triplicate and the results represented as the mean ± standard deviation. The IC_50_ values were calculated using a regression analysis. Statistical comparisons were made using one-way ANOVA, followed by Tukey’s post hoc test for multiple comparisons between the extracts (Prism GraphPad 8, GraphPad Software, Inc., San Diego, CA, USA). In this study, *p* values < 0.05 were considered statistically significant.

## 4. Conclusions

In this study a green extraction method for olive-leaf phenolics was developed using eco-friendly solvents consisting of PPG, LA, and water. Three extracts were prepared using the optimized extraction conditions and solvent mixtures, and their in vitro and anti-aging bioactivities were evaluated. The extracts were excellent antioxidants, as well as inhibitors of elastase, collagenase, tyrosinase, and lipoxygenase activity. The best activity was shown by the extracts prepared using magnetic-stirrer-assisted extraction for 20 min, using 0.8 g of herbal material suspended in 10 mL of PPG/LA/water mixture (28.6/63.6/7.8, *w*/*w*/*w*). Nevertheless, the sample prepared using the same extraction conditions but with 0.42 g herbal material was comparably active, and thus was the most ecologically acceptable extract. The investigated PPG/LA/water mixtures were active solvents that played an important role in the overall enzyme-inhibitory activity of the extracts. The prepared extracts were appropriate for direct use in cosmetic products, thus saving the time and energy necessary for the evaporation of conventional solvents.

## Figures and Tables

**Table 1:** Independent variables in the IV-optimal mixture design for magnetic-stirrer-assisted extraction* and content of total phenols (TP) and oleuropein in the extracts.

Standard	Run	X_1_ (%, *w*/*w*)	X_2_ (%, *w*/*w*)	X_3_ (%, *w*/*w*)	TP (mg/mL)	Oleuropein (mg/mL)
14	1	56.7	33.3	10.0	3.557	1.158
16	2	10.0	80.0	10.0	5.275	1.489
4	3	10.0	89.0	1.0	5.398	1.201
3	4	36.3	62.7	1.0	4.903	1.045
11	5	28.6	63.6	7.8	4.792	1.227
7	6	68.1	28.6	3.3	3.559	1.078
6	7	68.1	28.6	3.3	2.842	0.888
12	8	28.6	63.6	7.8	5.359	1.574
2	9	49.5	49.5	1.0	4.293	1.224
10	10	28.6	63.6	7.8	5.555	1.557
13	11	80.0	10.0	10.0	1.758	0.606
5	12	10.0	89.0	1.0	5.863	0.552
9	13	84.5	10.0	5.5	1.940	0.661
15	14	45.0	45.0	10.0	3.315	0.988
8	15	84.5	10.0	5.5	1.813	0.659
1	16	89.0	10.0	1.0	1.547	0.649

X_1_ = polypropylene glycol, X_2_ = water, X_3_ = lactic acid. * = magnetic-stirrer-assisted extraction, performed at 25 °C and 300 rpm.

**Table 2:** Analysis of variance (ANOVA) for the fitted model polynomial equations.

	TP					Oleuropein				
*R* ^2^	*R*^2^ = 0.9637, *R*^2^_P_ = 0.9456, *R*^2^_A_ = 0.9235					*R*^2^ = 0.8446, *R*^2^_P_ = 0.5741, *R*^2^_A_ = 0.7670				
Source	SS	DF	MS	*F-*Value	*p*-Value	SS	DF	MS	*F*-Value	*p*-Value
Model	351.0113	5	70.2023	53.1346	< 0.0001	1.0302	5	0.2060	10.8740	0.0009
Lack of Fit	6.0143	5	1.2029	0.8356	0.5757	0.0920	5	0.0184	0.9437	0.5246
Pure Error	7.1978	5	1.4396			0.0975	5	0.0195		

SS = sum of squares, DF = degrees of freedom, MS = mean square, *R*^2^_A_ = adjusted *R*^2^, *R*^2^_P_ = predicted *R*^2^, TP = total phenolic content.

**Table 3:** Comparison of the predicted and experimental values for the optimized solvents.

Solvent	Response	Optimization Goal	X_1_ (%, *w*/*w*)	X_2_ (%, *w*/*w*)	X_3_ (%, *w*/*w*)	*R*sp_pred_	*R*sp_obs_	RD (%)
A	TP (mg/mL)	Maximize	10.0	89.0	1.0	5.678	5.600	−1.4
B	Oleuropein (mg/mL)	Maximize	28.6	63.6	7.8	1.631	1.615	−1.0

X_1_ = polypropylene glycol, X_2_ = water, X_3_ = lactic acid, *R*sp_pred_ = predicted value, *R*sp_obs_ = observed value, RD = response deviation calculated as (*R*sp_obs_ − *R*sp_pred_) / *R*sp_pred_ × 100, TP = total phenolic content.

**Table 4:** Phytochemical composition of the prepared extracts.

Extract	TP (mg/mL)	TF (mg/mL)	TPA (mg/mL)	Oleuropein (mg/mL)	Verbascoside (mg/mL)
A08U	17.14 ± 1.01	0.38 ± 0.02	0.57 ± 0.02	3.46	0.81
B042M	11.52 ± 0.73	0.55 ± 0.03	0.73 ± 0.01	3.53	0.43
B08M	21.45 ± 1.72	0.98 ± 0.02	1.48 ± 0.05	6.47	0.76

TP = total phenolic content, TF = total flavonoid content, TPA = total phenolic acid content.

## Data Availability

Not applicable.

## Supplementary Materials

Table S1: Physicochemical properties of the prepared solvents; Table S2: Relationship between the concentrations of the target compounds and the HM/SR; Table S3: Volume of the prepared extracts; Table S4: Relationship between yield (%) of the target compounds and the HM/SR; Table S5: Relationship between the extraction parameters and the extended herbal extract/solvent ratio (HM/SR) (1.0–1.6 g/10 g solvent) in magnetic-stirrer-assisted extraction using solvent B.

## Sample Availability

Not available.
