# Supplementary material for: Optimization of Bioactive Phenolics Extraction and Cosmeceutical Activity of Eco-Friendly Polypropylene-Glycol–Lactic-Acid-Based Extracts of Olive Leaf
Source: Molecules. 2022 Jan 14;27(2):529. doi: 10.3390/molecules27020529 (PMC8778226; doi:10.3390/molecules27020529)
Supplement: Supplementary file 1 [file molecules-27-00529-s001.zip › molecules-1555609-supplementary.pdf]

Table S1. Physicochemical properties of the prepared solvents.

Standard	Run	ϱ (g/cm3)	η (Pas)	pH
14	1	1.040	0.0688	2.6
16	2	1.032	0.0031	1.83
4	3	1.014	0.0027	2.25
3	4	1.033	0.0145	2.68
11	5	1.045	0.0144	2.16
7	6	1.038	0.0603	3.13
6	7	1.032	0.0864	3.26
12	8	1.045	0.0144	2.16
2	9	1.038	0.0517	3.15
10	10	1.045	0.0144	2.16
13	11	1.035	0.1146	2.35
5	12	1.014	0.0027	2.25
9	13	1.020	0.0999	2.32
15	14	1.046	0.0984	2.5
8	15	1.020	0.0999	2.32
1	16	1.011	0.0835	2.31

Table S2. Relationship between the concentration of the target compounds and the HM/SR.

Content	Technique	Solvent	Equation	R^2
Total phenol	MSAE	A	y = 20.470××x + 1.100	0.9964
	UAE	A	y = 7.500××x + 1.241	0.9335
	MSAE	B	y = 28.250××x + 0.058	0.9825
	UAE	B	y = 11.190××x + 0.755	0.9920
Oleuropein	MSAE	A	y = 3.871××x + 0.344	0.9990
	UAE	A	y = 0.969××x + 0.392	0.9163
	MSAE	B	y = 7.779××x + 0.034	0.9919
	UAE	B	y = 6.247××x - 0.013	0.9976
Verbascoside	MSAE	A	y = 1.041××x - 0.008	0.9953
	UAE	A	y = 0.296××x + 0.068	0.9864
	MSAE	B	y = 0.922××x + 0.022	0.9997
	UAE	B	y = 1.319××x - 0.013	0.9937

MSAE = magnetic stirrer assisted extraction; UAE = ultrasound assisted extraction; Solvent A: 10% polypropylene glycol, 89% water, 1% lactic acid); Solvent B (28.6% polypropylene glycol, 63.6% water, 7.8% lactic acid). y = Concentration of the target compound (mg/mL), x = Herbal material/solvent ratio (g herbal material/10 g solvent).

Table S3. Relationship between yield (%) of the target compounds and the HM/SR.

Yield	Technique	Solvent	Equation	R^2
Total phenol	MSAE	A	y = 18.570××2 – 32.670×× + 20.420	0.9528
	UAE	A	y = 9.449××2 – 15.370××x + 8.498	0.9987
	MSAE	B	y = -5.640××2 – 5.408××x + 16.875	0.9366
	UAE	B	y = 4.907××2 – 13.120××x + 10.690	0.9928
Oleuropein	MSAE	A	y = 7.394××2 – 10.810××x + 5.194	0.9754
	UAE	A	y = -5.351××2 – 7.009××x + 2.610	0.9554
	MSAE	B	y = 2.934××2 – 6.511××x + 5.887	0.9668
	UAE	B	y = 3.238××2 – 5.971××x + 4.540	0.9464
Verbascoside	MSAE	A	y = 0.103××2 – 0.513××x + 0.631	0.9141
	UAE	A	y = 1.031××2 – 1.350××x + 0.530	0.9241
	MSAE	B	y = 0.501××2 – 1.004××x + 0.794	0.9800
	UAE	B	y = 0.795××2 – 1.337××x + 0.949	0.9564

MSAE = magnetic stirrer assisted extraction; UAE = ultrasound assisted extraction; Solvent A: 10% polypropylene glycol, 89% water, 1% lactic acid); Solvent B (28.6% polypropylene glycol, 63.6% water, 7.8% lactic acid). y = yield of the target compound (%), x = Herbal material/solvent ratio (g herbal material/10 g solvent).

Table S4. Relationship between the extraction parameters and the extended herbal extract/solvent ratio (HM/SR) (1.0-1.6 g/10 g solvent) in magnetic stirrer assisted extraction using the solvent B.

**Extraction parameter**	**Value of extraction parameter**	**HM/SR (g/10 g solvent)**	**Equation***	***R*²***
Total phenol content (mg/mL)	27.90	1.0	y = 25.090××x + 1.219	0.9912
	31.40	1.2		
	35.07	1.4		
	40.71	1.6		
Oleuropein content (mg/mL)	7.57	1.0	y = 7.737××x + 0.057	0.9967
	9.77	1.2		
	10.92	1.4		
	12.22	1.6		
Verbascoside content (mg/mL)	0.93	1.0	y = 0.956××x + 0.009	0.9982
	1.20	1.2		
	1.35	1.4		
	1.53	1.6		

*Includes the HM/RS values of 0.2-0.8. Extended weight of the herbal material (g) extracted by 10 g of the solvent –MSAE = magnetic stirrer assisted extraction; -UAE = ultrasound assisted extraction; Slovent A: 10% polypropylene glycol, 89% water, 1% lactic acid); Solvent B (28.6% polypropylene glycol, 63.6% water, 7.8% lactic acid). y = Concentration of the target compound (mg/mL), x = Herbal material/solvent ratio (g herbal material/10 g solvent).

Table S5. Volume of the prepared extracts.

Extraction technique	Solvent	HM/SR (g/10 g solvent)	V extract (mL)
MSAE	A	0.1	6.04
		0.2	5.27
		0.4	4.39
		0.6	3.54
		0.8	2.74
UAE	A	0.1	5.25
		0.2	3.57
		0.4	3.20
		0.6	3.02
		0.8	2.49
MSAE	B	0.1	6.34
		0.2	5.47
		0.4	4.98
		0.6	4.01
		0.8	3.19
		1.0	2.47
		1.2	1.85
		1.4	1.55
		1.6	1.26
UAE	B	0.1	5.94
		0.2	5.24
		0.4	4.50
		0.6	3.72
		0.8	2.77

MSAE = magnetic stirrer assisted extraction; UAE = ultrasound assisted extraction; Solvent A: 10% polypropylene glycol, 89% water, 1% lactic acid); Solvent B (28.6% polypropylene glycol, 63.6% water, 7.8% lactic acid).
